# 16SrDNA Pyrosequencing of the Mediterranean Gorgonian *Paramuricea clavata* Reveals a Link among Alterations in Bacterial Holobiont Members, Anthropogenic Influence and Disease Outbreaks

**DOI:** 10.1371/journal.pone.0067745

**Published:** 2013-06-26

**Authors:** Luigi Vezzulli, Elisabetta Pezzati, Carla Huete-Stauffer, Carla Pruzzo, Carlo Cerrano

**Affiliations:** 1 Department of Life, Earth and Environmental Sciences (DISTAV), University of Genoa, Genoa, Italy; 2 Department of Life and Environmental Sciences, Polytechnic University of Marche, Via Brecce Bianche, Ancona, Italy; UC Merced, School of Natural Sciences, United States of America

## Abstract

Mass mortality events of benthic invertebrates in the Mediterranean Sea are becoming an increasing concern with catastrophic effects on the coastal marine environment. Sea surface temperature anomalies leading to physiological stress, starvation and microbial infections were identified as major factors triggering animal mortality. However the highest occurrence of mortality episodes in particular geographic areas and occasionally in low temperature deep environments suggest that other factors play a role as well. We conducted a comparative analysis of bacterial communities associated with the purple gorgonian *Paramuricea clavata*, one of the most affected species, collected at different geographic locations and depth, showing contrasting levels of anthropogenic disturbance and health status. Using massive parallel 16SrDNA gene pyrosequencing we showed that the bacterial community associated with healthy *P. clavata* in pristine locations was dominated by a single genus *Endozoicomonas* within the order *Oceanospirillales* which represented ∼90% of the overall bacterial community. *P. clavata* samples collected in human impacted areas and during disease events had higher bacterial diversity and abundance of disease-related bacteria, such as vibrios, than samples collected in pristine locations whilst showed a reduced dominance of *Endozoicomonas* spp. In contrast, bacterial symbionts exhibited remarkable stability in *P. clavata* collected both at euphotic and mesophotic depths in pristine locations suggesting that fluctuations in environmental parameters such as temperature have limited effect in structuring the bacterial holobiont. Interestingly the coral pathogen *Vibrio coralliilyticus* was not found on diseased corals collected during a deep mortality episode suggesting that neither temperature anomalies nor recognized microbial pathogens are solely sufficient to explain for the events. Overall our data suggest that anthropogenic influence may play a significant role in determining the coral health status by affecting the composition of the associated microbial community. Environmental stressful events and microbial infections may thus be superimposed to compromise immunity and trigger mortality outbreaks.

## Introduction

Mass mortality events of benthic invertebrates from different phyla (sponges, cnidarians, molluscs, ascidians, bryozoans) occurred in recent years in the temperate Mediterranean Sea with catastrophic effects on the coastal marine ecosystem [1], [2]. Scientific evidence gathered so far indicated that sea surface temperature anomalies linked to global warming are among the primary triggering factors explaining such events [2], [3]. In particular, long and hot summer periods recorded during the recent years were observed to place a prolonged energetic constraints on benthic suspension feeders, which as a result, go into dormancy or decreased activity, leading to mortality in late summer and early fall [4], [5]. In addition nonresident thermodependant bacterial pathogens may take advantage both from high temperature and compromised host conditions contributing significantly to coral disease and mortality [6], [7]. For instance, we recently reported that *Vibrio*-TAV24 strain, belonging to the species *Vibrio coralliilyticus,* is involved in mass mortality events of the purple gorgonian *P. clavata* in the NW Mediterranean Sea [7].

In the above scenario, data coming from scleractinian corals suggest that alterations in bacterial holobiont members due to environmental stressors can disrupt coral’s immune system and enhance coral susceptibility to bacterial infections and diseases [8]. In fact, symbiotic bacteria are considered an important component of the “coral’s immune system” by preventing the colonization of nonresident, possibly pathogenic, bacteria [9]. Studies conducted on tropical corals have shown that, under stressful conditions, resident microbes critical to the healthy functioning of the coral organism are outcompeted by pathogenic microbes and usually in the context of environmental disruptions such as ‘heat waves’ during the warm summer months [10], [11].

However, despite acquired knowledge, we are still far from having a complete understanding of the causes triggering mass mortality events of benthic invertebrates in the Mediterranean Sea, especially concerning environmental stressors, other than temperature, which could be possibly involved. For example, occurrence of the mortality episodes is more frequent in some geographic areas of the Mediterranean Sea (e.g. Ligurian Sea and Adriatic Sea) compared to others, regardless of the seasonal temperature regime [12], [13]. In addition, mortality events were occasionally observed in the deep well below the thermocline layer [14]. The mesophotic zone comprised between 60 and 100–120 m depth is less subjected to climate driven changes than the upper ocean, and seasonal temperature follows lower fluctuations [15]. Clearly, as it appears from these considerations, additional factors other than seasurface temperature anomalies are likely to be involved in the occurrence of mortality events and still need to be addressed.

Impact by human activities, such as fisheries and recreational activities (e.g. diving), ship traffic and water pollution in coastal areas can lead local benthic populations to a general state of physiological stress. In particular, recent studies have shown that direct anthropogenic impact such as diving damage on *P. clavata* corals dramatically increase the natural mortality rate [16] as well as the time to quasi-extinction for populations subjected to realistic frequencies of mass mortality events [17]. Nevertheless, to date, little information is available on this subject, also possibly due to the fact that experimentally assessing the role of human factors in contributing to disease outbreaks is not trivial to pursue. For example, the use of laboratory model system (e.g. manipulative experiments based on coral growth in aquaria), largely employed in reductionist ecological studies, have a limited application to this specific purpose because unable to reproduce the complexity of the natural ecosystem and the global synergic influence of natural and anthropogenic factors in affecting coral health status in the field.

To overcome these concerns and explore additional factors possibly involved in the occurrence of mass mortality episodes in the Mediterranean Sea, we conducted a infield comparative study of bacterial communities associated with the purple gorgonian *P. clavata*, one of the most affected species, collected at different geographic locations and depth, showing contrasting levels of anthropogenic disturbance and health status. Our rationale considers the fact that alteration of the coral holobiont may be investigated as a proxy of coral health status and sensitivity to nonresident pathogens. The objective of this study was to assess whether anthropogenic influence affect the composition of the bacterial holobiont and if this could be linked to disease outbreaks.

## Materials and Methods

### Ethics Statement

The sampling in the Mediterranean Sea was performed in accordance with Italian laws. In particular for sampling in the Portofino and Tavolara marine areas permissions were granted by the “Marine Protected Area of Portofino (www.portofinoamp.it)” and the “Marine Protected Area Tavolara - Punta Coda Cavallo (http://www.amptavolara.com/en/home-page), respectively. For sampling in Pantelleria local competent authorities (Coast guard and Municipality of Pantelleria) were informed and allowed the sampling of biological material in non protected areas of the island for research purpose only. Italian laws do not ask for permissions for sampling in case of scientific purposes. This study did not involve endangered or protected species”.

### Study Areas

#### Portofino promontory (44°17′49′′N; 9°13′12′′E, Ligurian sea, Italy)

The cliff of Portofino Promontory (Ligurian Sea) is an oligocene puddingstone which reaches down to a depth of approximately 50 m. A large *P. clavata* population lives between 20 and 50 m deep. At the study site (“Punta del Faro”) population density is around 14 colonies/m2 [1]. Even if included in the general reserve of the Marine Protected Area (MPA) of Portofino, the study site is highly exploited with diving tourism and coastal sport fishing (trolling, casting, jigging), especially during summer season (about 5000 dives per year are estimated at the “Punta del Faro” diving site). Local people can be authorized to use also gill nets, and `palamiti’ (a type of long-lining, about 1 gear/day per 10 hectare). All these activities have a strong impact on the benthic communities especially in the upper layer (0–30 m).

#### Tavolara island (40°54′19′′N; 9°42′28′′E, Sardinia, NW Mediterranean sea, Italy)

The study sites of “Secca del Papa 1 and Secca del Papa 2” is a shoal facing the south-eastern side of Tavolara Island (North Sardinia). The site has a *P. clavata* distribution quite homogeneous, with a density ranging around 12 colonies/m^2^ [5]. The site is included in the general reserve of MPA of Tavolara and Punta Coda Cavallo. Professional fishing by residents of towns bordering the MPA within regulated weight limits by using environmentally sensitive equipment is permitted. Trawling and similar fishing techniques, and sport fishing are prohibited. Diving activities are here very frequent (about 5000 dives per year are estimated at the “Secca del Papa” diving sites).

#### Pantelleria island (36°49′28′′N; 12°0′52′′E, Mediterranean sea, Italy)

Pantelleria Island is unprotected but is not easily accessible being situated 70 km from the African coast and 85 km from Sicily. *P. clavata* populations show a scattered distribution, and are more common on the northward side of the island. At the study sites (“Cala Levante” and “Punta Spadillo”) coral populations have lower densities respect to Portofino and Tavolara sites. The highest frequency of lost fishing lines is in “Cala Levante”, one of the most accessible location of the island for local artisanal fishery. Regarding diving activities about 1000 dives per year are estimated at “Cala Levante” and 500 dives per year at “Punta Spadillo” diving sites (Spaggiari and Leonardi pers comm.).

### Experimental Design and Coral Sampling

**Figure 1:**
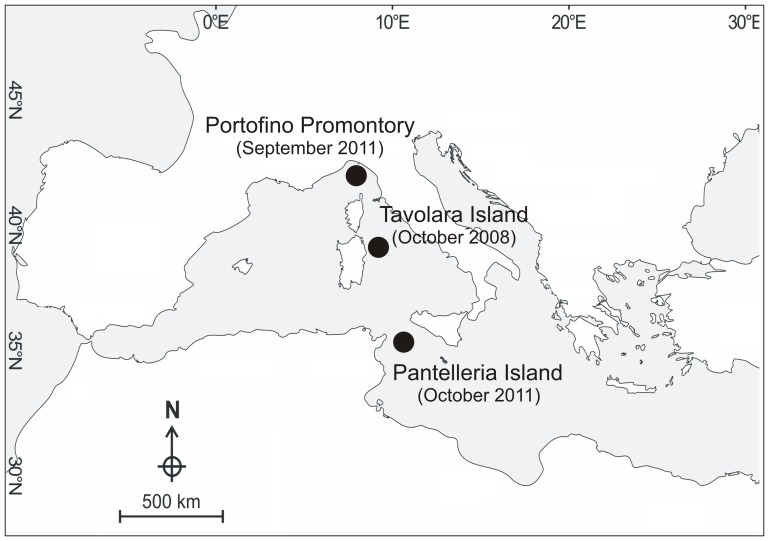
Geographic areas in the Mediterranean Sea sampled in this study.

**Figure 2:**
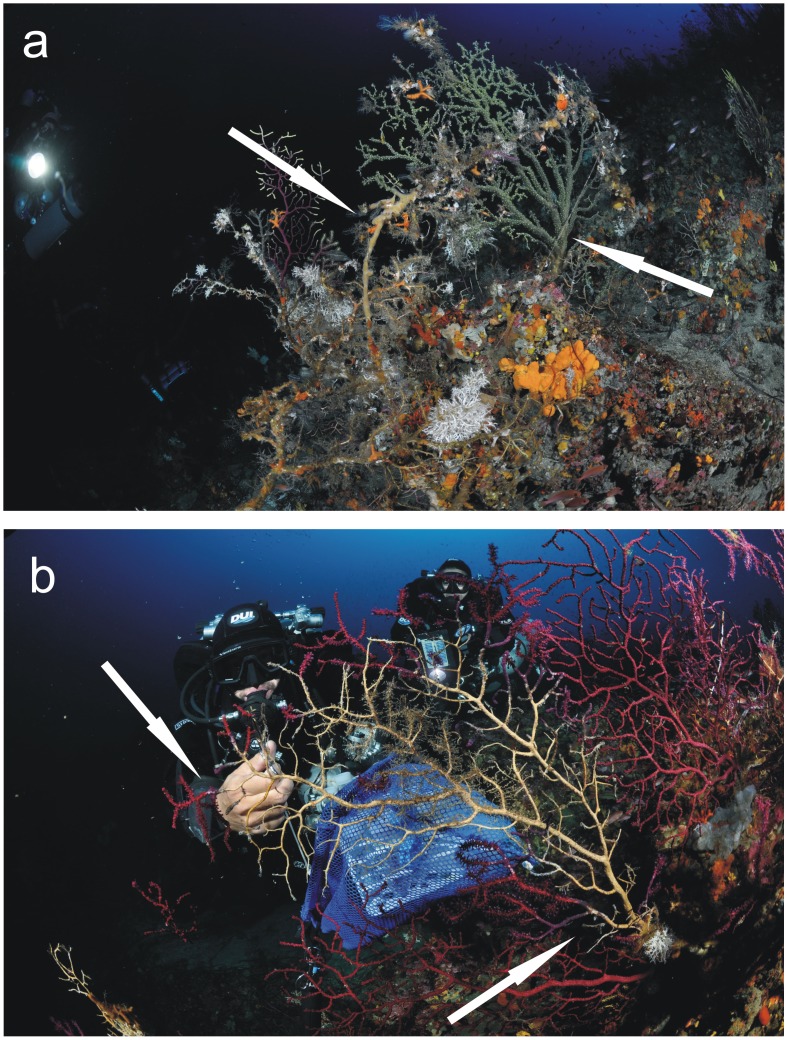
*P.* *clavata* population impacted by human activity. Human impacted *P. clavata* population sampled at “Cala Levante” (Pantelleria island) site in October 2011 at a depth of 63 m: (a) Coralligenous assemblage with a rope and a fishing line (arrows) entangling sea fans. The abrasive effect on sea-fan coenechime expose the bare skeleton to fouling organisms. (b) Sampling phase of a *P. clavata* colony with evident sign of necrotic tissue (arrows).

Samples of the coral *P. clavata* were collected at the different geographic areas (Portofino Promontory, Tavolara Island and Pantelleria Island) at different depths (euphotic and mesophotic) (**Fig. 1** **,** **Table 1**). In each area different coral populations were sampled at different sites showing contrasting level of anthropogenic influence (human impacted versus pristine populations, **Table 1**). In particular, human impacted populations were defined as those locally subjected to direct impact such artisanal fishery leading to mechanical injuries of the coral colonies (e.g. populations showing visible breakage of gorgonian branchings and high frequency of colonies entangled by fishing lines and nets) **(** **Fig. 2** **)**. The level of anthropogenic influence on coral populations was quantified by measuring for each diving spot the number of coral colonies entangled by fishing lines and nets wrapped around and reported into three ranking categories: absent = 0 entangled colonies per dive site (pristine populations); occasional = 1–5 entangled colonies per dive and frequent = >5 entangled colonies per dive (impacted populations) (**Table 1**). For each site the number of dives per year was also evaluated and reported into two ranking categories: low = <1000 dives/year; high >5000 dives/year (**Table 1**).

**Table 1:** *P. clavata* samples collected in this study.

Samples	Geographic area	Site	Date	Depth (m)	Level of Anthropogenic influence on coral populations		Coral Health status***
					Entangles colonies*	Number of dives**	
Pa30PH	PantelleriaIsland (Pa)	Punta Spadillo	October 2011	30	Absent (P)	Low	Healthy (H)
Ta30PH	TavolaraIsland (Ta)	Secca del Papa 2	October 2008	30	Absent (P)	High	Healthy (H)
Po50PH	PortofinoPromontory (Po)	Punta del Faro	September 2011	50	Absent (P)	Low	Healthy (H)
Pa90PH	PantelleriaIsland (Pa)	Punta Spadillo	October 2011	90	Absent (P)	Low	Healthy (H)
Po30IH	PortofinoPromontory (Po)	Punta del Faro	September 2011	30	Frequent (I)	High	Healthy (H)
Pa63ID	PantelleriaIsland (Pa)	CalaLevante	October 2011	63	Frequent (I)	High	Diseased (D)
Ta30ID	TavolaraIsland (Ta)	Secca del Papa 1	October 2008	30	Occasional (I)	High	Diseased (D)

Collected samples from different coral populations living in different geographic areas, at different depth and showing different health status condition. The level of direct anthropogenic Impact on coral populations at each site was quantified by measuring.

* the number of coral colonies affected by fishing lines and nets wrapped around: absent = 0 entangled colonies/dive (P-pristine); occasional = 1–5 entangled colonies/dive and frequent = >5 entangled colonies/dive (I-impacted).

** the number of dives per year is also reported: low = <1000 dives/year; high >5000 dives/year.

*** Diseased corals (D) are defined as those showing early symptoms of disease such as colonies displaying necrotic coenenchyme (pale pink to grayish colour) and patchy tissue loss exposing bare areas of the skeletal axis [7]. Healthy corals (H) are defined as those with no apparent symptoms of disease displaying a purple coloration of the coral tissue [7].

At “Secca del Papa 1” (Tavolara island) and “Cala Levante” (Pantelleria island) sites sampled populations were affected by ongoing mortality episodes and showed early symptoms of disease (**Table 1**). In particular, at Tavolara Island in October 2008 a mortality episode occurred between 20 m to 40 m depth with an high percentage of affected colonies, which was close to 100% at a depth of 25 m [5]. At Pantelleria Island in October 2011 necrotic colonies were observed below 60 m depth. Percentage of affected colonies at this site was estimated less than 10% of the local population. At both sites disease symptoms observed on coral populations matched those previously recorded during *P. clavata* mortality episodes in the NW Mediterranean Sea [1], [2] with coral colonies displaying necrotic coenenchyme (pale pink to grayish colour contrasting the purple colouration of healthy tissue) and patchy tissue loss exposing bare areas of the skeletal axis [7].

At “Punta del Faro” (Portofino promontory) strong massive mortalities of *P. clavata* were reported in 1999, 2003 [1], [2] and albeit not observed during the course of the present study necrotic colonies are detectable very often at the end of each summer season.

Branch fragments (n = 5) of *P. clavata* (5 cm length) from different colonies were collected by scuba divers using sterile cutters and placed into sterile disposable 50 ml polypropylene tubes underwater. The use of SCUBA TRIMIX technology allowed the collection of coral samples from the deep mesophotic layer and for the first time during a *P. clavata* deep mortality event (63 m, deep in relation to previously described mortalities) at Pantelleria Island. Diseased samples were taken from colonies showing early stages of lesions without tissue loss to avoid the bias of later bacterial secondary colonization [7]. In the laboratory, samples were gently washed three times with 50 ml of artificial seawater (ASW) to remove non-associated bacteria. Coral samples were then stored in RNALater solution (Life Technologies) at room temperature.

### Nucleic Acid Extraction

After removing RNA later solution approximately 1 g (wet weight) of coral tissue was detached from coral skeleton by scraping and crushed. DNA was extracted from tissue using the High Pure PCR template preparation kit (Roche Diagnostics, Mannheim, Germany) following the manufacturer’s instructions. The amount of DNA extracted was determined fluorimetrically with QuantiFluor™ dsDNA System using a QuantiFluor™fluorometer (Promega Italia srl, Milano, Italy).

### Real-Time PCR

The *Vibrio* relative abundance index (VAI) was calculated as previously described using 16S rRNA gene targeted real-time PCR with SYBR-green detection using a capillary-based LightCycler instrument (Roche Diagnostics, Mannheim, Germany) and a standard curve method for quantification [18]. Briefly10 ng of genomic DNA extracted was used. The oligonucleotide primers for the PCR reaction were Vib1 f-5′-GG CGTAAAGCGCATGCAGGT-3′ and Vib2 r-5′-GAAATT CTACCCCCCTCTACAG-3′ [19], specific for the genus *Vibrio*, and 967f-5′-CAACGCGAAGAACCTTACC-3′ and 1046r-5′-CGACAGCCATGCANCACCT-3′ [20], specific for the domain Bacteria, amplifying positions 567–680 and 965–1063 (V6 hyper variable region) of the *Escherichia coli* numbering of the 16S rRNA, respectively. Each reaction mixture contained 5.0 mmol of MgCl2 and 0.25 mmol of each primer in a final volume of 20 µl. The PCR programme was optimised as follows: initial denaturation at 95°C for 10 min, subsequent 40 cycles of denaturation at 95°C for 5 s, annealing at 58°C (*Vibrio* spp.) or 57°C (total bacteria) for 5 s and elongation at 72°C for 4 s, followed by a final elongation at 72°C for 10 min. PCR runs were analyzed directly in the LightCycler using melting analysis and the software provided with the instrument. For each single real-time PCR assay each DNA template was analysed in triplicate (coefficient of variation <5%). The standards were prepared from 16S rDNA nucleic acid templates of *V. cholerae* El Tor N16961 at known molar concentrations. *Vibrio* spp. and the total bacterial concentrations were expressed as number of cells per g of samples by dividing the total 16SrDNA copy number by the average 16SrDNA copy number in vibrios (n = 9, [21]) and proteobacteria (n = 3.5, [22]), respectively.

For the direct detection of the species *V. coralliilyticus* in coral samples species-specific primers (dnaJF ATATGGTCATGCAGCCTTC; dnaJR GCGAACCGCTTCTTCTAGT, [18] and vcpARTF AGCTACGACTGCCGCCCTTAC; vcpARTR-GGAGCCCTTTCACTTACGATG TTG, [23]) were used. PCR conditions were set as on the above with annealing temperature of 59°C (dnaJ primers) or 60°C (vcpART primers). Amplicons generated were also checked by agarose [gel] electrophoresis, purified with spin columns (Roche Diagnostics, Mannheim, Germany) and sequenced using the automated ABI Prism 3730 DNA sequencer (Applied Biosystems).

### 16S rDNA Pyrosequencing

A 16SrDNA PCR amplicon library was generated from genomic DNA extracted and pooled from five replicate coral samples using the broad-range bacterial primers, 967f and 1046r, amplifying the V6 hypervariable region of ribosomal RNAs [20]. The PCR products were pooled after cycling and cleaned to a total yield of 300 ng using an AMICON Ultra 30K membrane (Millipore, Billerica, MA, USA). Amplicon libraries were bound to beads under conditions that favour one fragment per bead and beads were emulsified in a PCR mixture in oil. After breaking the emulsion, the DNA strands were denatured, and the beads carrying single-stranded DNA clones were deposited into the wells on a PicoTiter- Plate (454 Life Sciences, Branford, CT, USA) for pyrosequencing on a 454 GS Junior System (Roche, Basel, Switzerland). Sequence reads data were archived at NCBI Sequence Read Archive (SRA) with accession number: SRP020949.

### Bioinformatics

The open-source, platform-independent, community-supported software program, mothur [24], was used to process and analyze the sequence data. Sequence reads were trimmed as described (see Results and Discussion) and chimeric sequences were removed. To assess abundance and phylogenetic identity, each trimmed read sequence was BLASTed against a reference database of nearly 400.000 rRNA genes for the bacterial domain available from the SILVA rRNA database project (http://www.arb-silva.de/). MEGAN (Metagenome Analysis Software, version 3.8, University of Tübingen, Tübingen, Germany) analysis was then carried out using a bit-score threshold of 35 and retaining only hits whose bit scores were within 10% of the best score. In addition all assignments hit by <20 reads were discarded.

To assess taxonomic diversity sequences were clustered as operational taxonomic units (OTUs) at 97% similarity. OTU diversity was examined using biodiversity indices and rarefaction analysis by the mothur software.

### Statistical Analysis

Agglomerative hierarchical clustering (CLUSTER analysis), with the unweighted pair-group average cluster model, was applied to group together coral samples of similar taxonomic composition. In addition non-Metric multi-Dimensional Scaling (nMDS) ordinations derived from BrayeCurtis similarity matrices, were used to visualize differences in the overall structure of microbial assemblage associated with corals. Cluster and ordination analyses were performed with the software PRIMER 6 [25]. Three-way Analysis of Variance (ANOVA) was used to test differences in average VAI values among *P. clavata* samples collected in different geographic areas (Portofino promontory vs Tavolara island vs Pantelleria island) at different depth (photic vs mesophotic) and showing different health status condition (healthy vs diseased). ANOVA test was performed using the MATLAB Statistics Toolbox (Version 6.1; TheMathWorks). Data for all statistics were square root transformed prior to the analysis.

## Results and Discussion

### Overall Bacterial Diversity and Community Structure Associated with the Purple Gorgonian *P. clavata* in the Mediterranean Sea

We sequenced 133.853 PCR amplicons spanning the V6 hypervariable region of 16SrDNA gene from *P. clavata* genomic DNA extracted from different coral populations living in different geographic areas, at different depth and showing different health status condition (**Table 1**). The number of reads per sample ranged from 10.642 to 15.240 sequences. To minimise the bias associated to random sequencing errors, a stringent trimming procedure was conducted, by eliminating reads that contained one or more ambiguous bases, had errors in the barcode or primer sequence, were atypically short (<70 bp), and had an average quality score <30 [20]; on average this step reduced the size of the data set by 15%.

**Figure 3:**
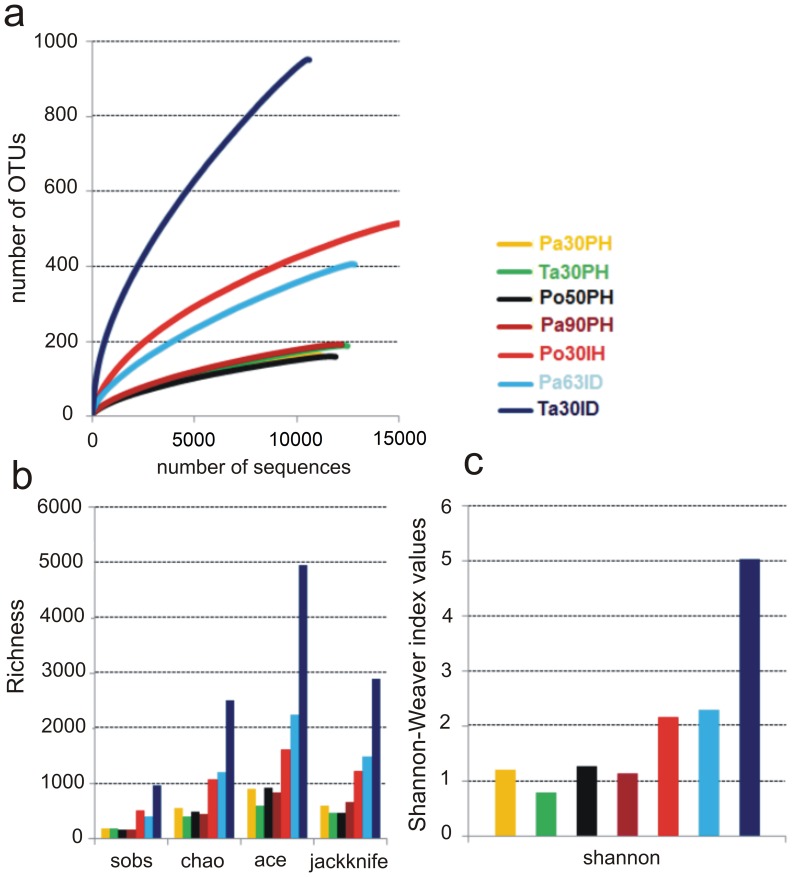
Bacterial diversity associated with *P.* *clavata*. Alpha diversity metrics derived from 16S rDNA pyrosequencing of bacteria associated with *P. clavata* samples: (a) Rarefaction curves; (b) number of OTUs predicted (Chao1, Ace, jackknife) and observed (sobs); (c) Shannon-Weiner diversity of bacteria from each coral sample. OTU’s were grouped at >97% similarity based on mothur classified results.

Species richness of the bacterial communities associated with *P. clavata* was highest in coral samples collected from populations subjected to anthropogenic disturbance and disease events such as those of Po30IH, Pa63ID, and Ta30ID samples, whilst declined to less than half in corals collected from pristine populations (samples Pa30PH, Ta30PH, Po50PH, Pa90PH) (**Fig. 3**). The bacterial diversity associated with *P. clavata* estimated by the Shannon diversity index from the OTU data was also high for human impacted samples varying from 2.2 to 5.0 with an average of 3.2 (SD = 1.6). In contrast, diversity was lower in coral samples collected in pristine areas that showed comparable values of the Shannon index ranging from 0.8 to 1.3 with an average of 1.1 (SD = 0.2) **(** **Fig. 3** **)**. In these latter rarefaction curves, analysis revealed that the diversity had almost reached a stable value **(** **Fig. 3** **)**. In contrast, in coral samples from human impacted populations, rarefaction curves showed a diverse pattern, which failed to reach a plateau. This was evident in Po30IH, Pa63ID, and Ta30ID samples suggesting that a large number of unseen OTUs still existed in the original samples and more sequencing effort should be required to detect additional phylotypes. According to this, the number of OTUs in these samples estimated by ACE, Chao1 and jackknife richness estimator was considerably higher than the number of observed OTUs (sobs) representing less than 50% of the estimated richness (**Fig. 3**). This means, for example, that for OTU numbers generated by the Chao1 estimator there were between 546 and 1550 additional phylotypes with a final richness of between 1069 and 2501 in each sample. Considering the current sequencing effort, discordance between estimated and observed richness in these samples may be due to the effects of rare species as it is well know that microbial communities naturally contain a large number of rare species and a small number of abundant ones [20].

To investigate most abundant members of the coral holobiont phylogenetic identity of generated sequences from coral samples were evaluated using the Metagenome Analysis Software (MEGAN). Each trimmed read sequence was BLASTed against a reference database of nearly 400.000 rRNA genes for the bacterial domain as described in the method session. The results of BLASTN were then used to estimate the taxonomic content of the data set, using NCBI taxonomy with MEGAN [26]. Only the most abundant reads (those occurring at least 20 times in the trimmed data set) were assigned to bacterial taxa and included in the results. Using this threshold and quality filters described in the method session the lowest common ancestor algorithm assigned 85 231 reads to the domain Bacteria, while 26 remained unassigned, either because the bit-score of their matches or the minimum number of reads for taxon assignment fell below the threshold.

Bacterial community were dominated by the class of *Gammaproteobacteria* that accounted on average for >95% of the overall community structure in healthy corals from pristine populations. Among this class the most dominant bacterial genus in the microbiome of *P. clavata* was *Endozoicomona*s within the order *Oceanospirillales* which represented ∼90% of the overall bacterial community. This genus is frequently found in association with marine invertebrates and the type strain *Endozoicomonas elysicola* was firstly isolated from the sea slug *Elysia ornata* off the coast of Izu-Miyake Island, Japan [27]. The species *E. montiporae* was later reported from the encrusting pore coral *Montipora aequituberculata* in Taiwan [28]. Clade of bacteria in the *Oceanospirillaceae* is also widely distributed in *Porites* spp. and other hermatypic corals [29]. Interestingly, it has been recently shown that *Endozoicomonas* represents the dominant bacterial genus associated to the Mediterranean gorgonian coral *Eunicella cavolinii*, a species closely related to *P. clavata* [30]. Other bacterial classes consistently found in the microbiome of *P. clavata* were alphaproteobacteria (∼5%), betaproteobacteria (∼0.2%), and actinobacteria (∼0.3%). The genus *Vibrio* which includes species pathogenic for corals accounted on average for less than 1% of the healthy coral bacterial community.

### Comparative Analysis of the Bacterial Communities Associated with *P. clavata* Corals Collected from Different Geographic Areas and Showing Contrasting Levels of Anthropogenic Disturbance and Health Status

**Figure 4:**
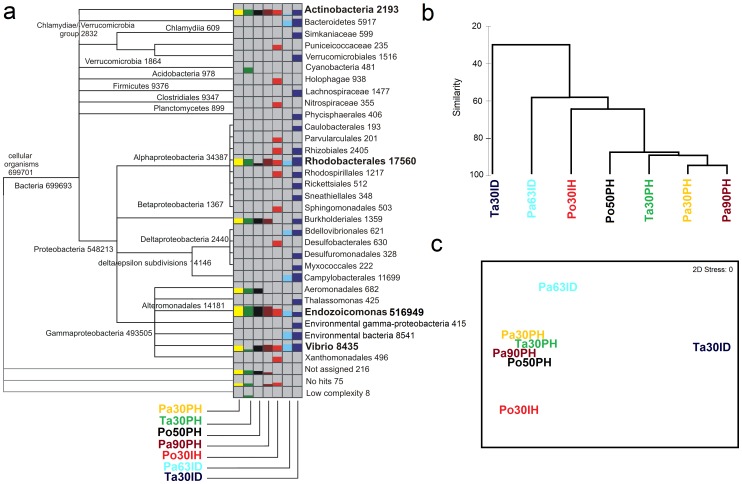
Comparative analysis of the bacterial communities associated with *P.* *clavata*. 16S rDNA pyrosequencing-based comparative analysis of dominant bacterial groups associated with *P. clavata* samples collected in different geographic areas, at different depth and showing different health status condition. (a) a comparative map is shown, where the numbers of normalised reads taken by each taxon (the tree is collapsed to the ‘genus’ level) in each year are represented as colour bar. The cumulative number of normalised reads across the different coral samples is also shown for each taxon [29]. Genus shared across all samples are in bold (b) agglomerative hierarchical clustering (CLUSTER analysis) and (c) non-Metric multi-Dimensional Scaling (nMDS) of the different sample datasets.

We used a multiple-comparative analysis in MEGAN to compare the bacterial communities associated with *P. clavata* corals collected as previously described **(** **Table 1** **)**. To minimize potential bias arising from differences in absolute read counts taxon data were normalised over all reads, such that each data set had 100 000 reads [31]. As detected by OTU diversity analyses we observed that shifts in bacterial assemblages associated with *P. clavata* occurred mainly in relation to the level of human impact and health status of the coral samples. Accordingly Po30IH, Pa63ID and Ta30ID samples were clearly separated from other samples by CLUSTER and MDS analyses (**Fig. 4**). Interestingly, a relative decrease in the dominant bacterial symbionts *Endozoicomonas* spp. was observed in these samples. In particular, contribution by *Endozoicomonas* genus to the overall bacterial community decreased from more than 90% dominance in *P. clavata* samples from pristine populations (Pa30PH, Ta30PH, Po50PH, Pa90PH) to less than 70% dominance in Po30IH and Pa63ID to 5% in Ta30ID. In contrast, bacterial symbionts exhibited remarkable stability in *P. clavata* collected at euphotic and mesophotic depth in pristine locations (e.g. Pa30PH and Pa90PH samples, dominance >90%) suggesting that fluctuations in environmental parameters such as irradiance and temperature (typically exhibiting strong seasonal variations in the surface euphotic layer and almost constant low values in the deep mesophotic layer) have limited effect in structuring the bacterial holobiont.

The decrease in dominance by *Endozoicomonas* members of the bacterial holobiont in diseased *P. clavata* samples (Pa63ID and Ta30ID) and in samples collected in shallow environments at Portofino Promontory (Po30IH), one of the most affected area in the Mediterranean Sea by recurring mortality events [32] suggests that this dominant genus may contribute to the health status of the coral. Accordingly, coral symbionts are thought to provide benefit to their hosts by supplying food compounds such as organic carbon, nitrogen or secondary metabolites [33]. In particular, it has been recently shown that member of the order of *Oceanospirillales* can utilize constituents of coral tissues and mucus such as organic acids, amino acids, ammonium, and dimethylsulfoniopropionate (DMSP) [34]. High concentrations of DMSP and DMS have been found within animals that harbor symbiotic algae, such as scleractinian corals, providing a potential link between DMSP-degrading *Oceanospirillales* and corals [35], [36]. Nevertheless, the functional and ecological role of the dominant *Endozoicomonas* genus in the azooxanthellate purple gorgonian *P. clavata* is still largely unknown. Microbial symbionts may also protect corals from disease, for instance by producing antimicrobial chemical defenses targeted at pathogens or other potentially deleterious microorganisms and by preventing unwanted microbial colonization through the occupation of otherwise available niches [37], [38], [39].

Accordingly, the study of the *Vibrio* community in our samples clearly showed that VAI index was significantly higher in diseased than in healthy corals concomitant with the decrease of *Endozoicomonas* spp. (ANOVA, p<0.05, **Table S1**). In addition, bacterial taxa such as *Bacteroidetes*, *Bdellovibrionales*, *Campylobacterales* as well as unclassified environmental bacteria were only found in diseased animals although their possible role in coral pathogenesis is unknown **(** **Fig. 4** **)**.

**Figure 5:**
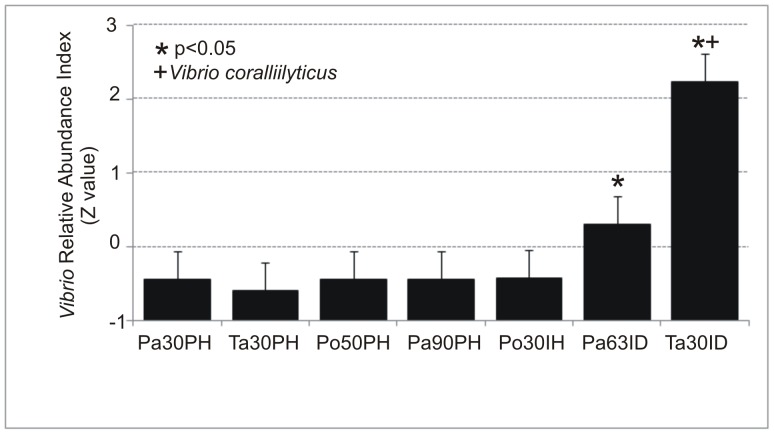
Analysis of *Vibrio* populations. *Vibrio* relative abundance index (VAI) calculated on *P. clavata* samples collected in different geographic areas, at different depth and showing different health status condition. Z values are obtained by subtracting the population mean and dividing the difference by the s.d. * ANOVA p<0.05;+presence of the species *V. coralliilyticus*.

Vibrios are a large group of indigenous marine bacteria with over 80 species described, also including several species capable of causing infection in humans and animals [40]. In a previous study we recently conducted to assess the potential role of microbial pathogens in contributing to mass mortality events of *P. clavata* in the NW Mediterranean Sea, we observed that *Vibrio* species are generally pathogenic toward *P. clavata* corals being capable to produce tissue damage during experimental infection conducted in aquaria [7]. In particular, we described a TAV24 strain later identified as *V. coralliilyticus* which satisfied Koch’s postulates and was linked thus to coral disease observed during mortality episodes [7]. The TAV 24 strain is a non resident pathogen as in a larger survey conducted from 2008 to 2011 in three different areas of the NW Mediterranean Sea affected by mortality episodes (Portofino Promontory, Tavolara Island and Capo Mortola Marine Reserve) it was consistently found in association to disease corals but never found in healthy corals (data not shown). Interestingly although *V. coralliilyticus* was detected on diseased corals collected at Tavolara Island in 2008 it was not found on diseased corals collected during a deep mortality episodes occurred at Pantelleria Island in 2011. To our knowledge, this is the first study investigating a deep mortality episode of *P. clavata* occurred in the mesophotic layer of the Mediterranean Sea **(** **Fig. 2** **)**. Temperature recorded at this depth is almost constant all year round and rarely exceed 18°C which can be taken as a threshold above which physiological stress and microbial proliferation could occur in *P. clavata* [7] [41]. This suggests that neither temperature anomalies nor recognized microbial pathogens such as *V. coralliilyticus* TAV24 strain which are currently considered among the main casual factors triggering mortality outbreak in *P. clavata* populations are solely sufficient to fully explain the occurrence of these events. As a speculation the increased dominance of *Vibrio* spp. (non-*V. coralliilyticus* species*)* observed in Pa63ID samples compared to healthy coral samples may have contributed to disease as well as the presence of yet unrecognized pathogens. It is, nevertheless, worth mentioning that infection by TAV24 strain was associated to a more severe disease event at Tavolara (almost 100% of affected colonies) than at Pantelleria (less than 10% of affected colonies) where the strain was not found. Accordingly the alteration of the bacterial holobiont members (e.g. increased specie richness and diversity, **Fig. 3**) and dominance of *Vibrio* spp. (**Fig. 5**) was greater in Ta30ID than Pa63ID. *Vibrio* species may thus take advantage of the shift in bacterial holobiont members and, when conditions are favorable (e.g. high sea surface temperature) possibly trigger the occurrence of diseases. The same conditions may favors coral infection by highly virulent strains, such as TAV24 strain, that may be responsible for larger disease outbreaks such as the one occurred at Tavolara island in October 2008.

### Conclusions

Overall our data suggest that influence by direct human activities, such as the mechanical injuries considered in the present study, may play a significant role in determining the coral health status by leading to a more unstable microbial community (e.g. altering the composition and diversity of the associated bacterial community). Changes in the structure of coral associated microbial communities such as the ones observed in healthy coral populations affected by direct anthropogenic influence (Po30IH) and similarly, in diseased corals (Pa63ID, Ta30ID) under comparable human pressure, point to a link among alteration in bacterial holobiont members, direct human influence and susceptibility of corals to microbial pathogens and associated diseases. According to this scenario, a decrease in the contribution of dominant *Endozoicomonas* spp. in *P. clavata* from populations subjected to human disturbance might possibly explain for the colonization of disease-related bacteria such as vibrios, and/or other not yet recognized pathogens, by the opening of new available niches. Environmental and climate linked stressful events such as the occurrence of temperature anomalies and starvation periods as well as the presence of highly virulent microbial strains may thus be superimposed to compromise immunity and trigger mortality outbreaks.

## Supporting Information

Table S1 Output of the three-way Analysis of Variance (ANOVA) applied to test differences in average VAI values among *P. clavata* samples collected in different geographic areas (Portofino promontory vs Tavolara island vs Pantelleria island) at different depth (photic vs mesophotic) and showing different health status condition (healthy vs diseased).
(DOCX)
