# Supplementary material for: 16SrDNA Pyrosequencing of the Mediterranean Gorgonian Paramuricea clavata Reveals a Link among Alterations in Bacterial Holobiont Members, Anthropogenic Influence and Disease Outbreaks
Source: PLoS One. 2013 Jun 26;8(6):e67745. doi: 10.1371/journal.pone.0067745 (PMC3694090; doi:10.1371/journal.pone.0067745)
Supplement: Table S1 — Output of the three-way Analysis of Variance (ANOVA) applied to test differences in average VAI values among P. clavata samples collected in different geographic areas (Portofino promontory vs Tavolara island vs Pantelleria island) at different depth (photic vs mesophotic) and showing different health status condition (healthy vs diseased). (DOCX) [file pone.0067745.s001.docx]

| Source | **Sum Sq.** | **d.f.** | **Mean Sq.** | **F** | **P** |
| --- | --- | --- | --- | --- | --- |
| Geographic areas | 0.040 | 2 | 0.020 | 1.70 | 0.21 |
| Depth | 0.017 | 1 | 0.017 | 1.47 | 0.24 |
| Health status | 0.785 | 1 | 0.785 | 66.86 | 0.01 |
| Error | 0.188 | 16 | 0.012 |  |  |
| Total | 1.378 | 20 |  |  |  |
